# Olive Oil Lipophenols Induce Insulin Secretion in 832/13 β-Cell Models

**DOI:** 10.3390/pharmaceutics13071085

**Published:** 2021-07-16

**Authors:** Maria Cristina Caroleo, Pierluigi Plastina, Alessia Fazio, Chiara La Torre, Fabrizio Manetti, Erika Cione

**Affiliations:** 1 Department of Pharmacy, Health and Nutritional Sciences, University of Calabria, 87036 Rende, Italy; mariacristinacaroleo@virgilio.it (M.C.C.); pierluigi.plastina@unical.it (P.P.); alessia.fazio@unical.it (A.F.); latorre.chiara@libero.it (C.L.T.); 2 Department of Biotechnology, Chemistry and Pharmacy, University of Siena, Via Aldo Moro 2, I-53100 Siena, Italy

**Keywords:** lipophenols, FFAR1, GSIS, insulin secretion, hydroxytyrosol, tyrosol, polyphenol

## Abstract

Glycemic control is a mainstay of type 2 diabetes mellitus (T2DM) clinical management. Despite the continuous improvement in knowledge and progress in terms of treatment, the achievement of the physiologic metabolic profile is still an ongoing challenge in diabetic patients. Pancreatic β-cell line INS-1 832/13 was used to assess the insulin secretagogue activity of hydroxytyrosyl oleate (HtyOle) and tyrosyl oleate (TyOle), two naturally occurring lipophenols deriving from the conjugation of oleic acid (OA) and hydroxytyrosol (Hty) or tyrosol (Ty), respectively. The insulin secretion was determined under a glucose-induced insulin secretion (GSIS) condition by the ELISA method. The potential involvement of G-protein-coupled receptor 40 (GPR40), also known as free fatty acid receptor 1 (FFAR1), was investigated by both molecular docking and functional pharmacological approaches. Herein, we demonstrated that HtyOle and TyOle exerted a facilitatory activity on insulin secretion under the GSIS condition. Moreover, we provided evidence that both lipophenols are natural modulators of FFAR1 receptor. From our results, the anti-diabetes properties associated with olive oil consumption can be partly explained by the HtyOle and TyOle effects.

## 1. Introduction

Type 2 diabetes mellitus (T2DM) is one of the most common disorders seen in clinical practice; it affects about 9.6% of adults in the world [1], leading to a poor quality of life and a higher risk of developing co-morbidities. The condition stems from deficient insulin secretion from pancreatic β-cells and the occurrence of insulin resistance in peripheral tissues [2]. All therapeutic approaches for T2DM are aimed at decreasing blood glucose levels, since optimal metabolic control delays the onset and slows the progression of cardiovascular complications. Several glucose-lowering agents are available in clinical practice; however, only about half of type 2 diabetic patients achieve glycemic control, and the occurrence of unwanted side-effects often hampers the use of these medications [3]. Therefore, there is an urgent need for more efficient and safer therapeutic options. In that context, an interesting antidiabetic profile has been shown by dietary phytochemicals [4] and attention has been paid to extra virgin olive oil (EVOO) owing to its ability to decrease gastrointestinal levels of glucose, as well as the natural hormones, incretins [5,6]. Usually, the high content of monounsaturated fatty acids (MUFAs), particularly in oleic acid (OA, C18:1), where the percentage ranges from 55 to 83%, is regarded as the major determinant in the EVOO beneficial effects [7]. Indeed, meta-analyses of randomized controlled trials have pointed out a net improvement of metabolic parameters in T2DM patients after replacing carbohydrates (about 5–10% of total energy intake) with MUFAs [8,9,10]. Of note, OA is a natural ligand of G-protein-coupled receptor 40 (GPCR-40) [11], a member of the GPCR family of seven-transmembrane domain proteins that couple extracellular stimuli to intracellular responses, mainly via the heterotrimeric G proteins and β-arrestins [12]. GPCRs are involved in a variety of biological processes, including the fine tuning of Langerhans islets and enteroendocrine cell activities [13]. GPR40, also known as free fatty acid receptor 1 (FFAR1), is predominantly found at the level of pancreatic β-cells [13,14]. Deletion of this receptor significantly decreases the potentiation of GSIS by fatty acids, both in vivo and in isolated islets [15], indicating that FFAR1 is required for normal insulin secretion. Of note, FFAR1 is also highly expressed at the level of enteroendocrine cells, where it induces glucagon-like peptide-1 secretion, thus avoiding hypoglycemic risk [16]. The tissue distribution and functional role make FFAR1 an attractive therapeutic target for T2DM. Several synthetic molecules targeting FFAR1 have been developed using high-throughput screening and subsequent medicinal chemistry approaches, including the ago-allosteric modulator TAK-875 (Fasiglifam) or receptor agonists such as GW9508, AMG-837, and LY2881835. The beneficial effects of synthetic agonists to FFAR1 on glucose homeostasis in preclinical models have prompted a number of clinical trials [13]. Unfortunately, the results have been unsatisfactory, raising serious concerns about the safety profile of these compounds. In this frame, several different fatty acids (FAs) showed ‘extraordinarily flat structure–activity relationships (SAR)’ toward FFAR1 [17,18], and both experimental and theoretical findings have provided support for at least three different ligand-binding sites on the receptor [19,20]. Activation of the receptor by medium- and long-chain fatty acids provokes insulin release via the Ca^2+^-mobilizing G-protein Gαq [21]. In particular, OA through FFAR1 potentiates the second phase of GSIS, with a concomitant fast FFAR1-dependent F-actin depolymerization and activation of protein kinase D [22]. Among FAs, OA has received great interest because of its multiple beneficial effects on metabolic balance, although it promotes insulin secretion at the lipotoxic concentration of 100 μM [23,24]. On the other hand, phenolic compounds are known to contribute to EVOO health benefits, even if they represent a minor fraction in the composition of olive oil [25]. In this context, hydroxytyrosol (Hty) and tyrosol (Ty) have been shown to preserve β-cell function and survival, and to exert anti-diabetic effect in animal models of T2DM [26,27]. However, the polar nature of Hty and Ty and their unfavorable pharmacokinetic profiles has hampered the use of these compounds in humans. The synthesis and the evaluation of Hty and Ty conjugates with fatty acids was proposed in order to improve the lipophilic nature and pharmacokinetic properties of Hty or Ty themselves [28,29,30]. It is worth noting that some of these lipophenols, namely hydroxytyrosyl and tyrosyl oleate (esters of OA with Hty or Ty, HtyOle and TyOle, respectively), represent a significant form in which Hty and Ty naturally occur, as HtyOle and TyOle were identified in olives and olive oil, as well as in the by-products of olive oil production [31,32,33]. In the present study, we investigated the role of HtyOle and TyOle on insulin secretion in the INS-1 832/13 cell line model under the GSIS condition. We found that the insulin secretagogue effects of these compounds were achieved at concentrations ranging from 10 to 40 µM. To gain further insight into the underlying molecular mechanism, we performed functional pharmacological tests and molecular docking study simulation. The latter is crucial to predict the preferred orientation of HtyOle and TyOle and the strength of association between HtyOle or TyOle and the FFAR1 receptor.

## 2. Materials and Methods

### 2.1. Chemicals and Reagents

Tyrosol (Ty), oleic acid (OA), methyl oleate, *t*-butanol, *n*-hexane, acetone, and dimethyl sulfoxide (DMSO) were supplied by Sigma–Aldrich (Milan, Italy). The Novozym^®^435 (immobilized *Candida antarctica* Lipase B) was from Novozymes (Bagsværd, Denmark). The aqueous solutions were prepared using ultrapure water, with a resistivity of 18.2 MΩ cm, obtained from a Milli-Q plus system (Millipore, Bedford, MA, USA). Roswell Park Memorial Institute 1640 (RPMI-1640) medium, fetal bovine serum (FBS), l-glutamine, and penicillin/streptomycin were obtained from Thermo Fisher Scientific (Waltham, MA, USA).

### 2.2. Synthesis of Hty, HtyOle and TyOle

Hty was synthesized as previously described [34]. The synthesis of HtyOle and TyOle was carried out according to our enzymatic method [31]. Briefly, hydroxytyrosol or tyrosol (1.6 mmol) were allowed to react in an orbital shaker at 50 °C with methyl oleate (3.2 mmol), in the presence of immobilized *Candida Antarctica* Lipase B (CALB, 200 mg), in *t*-butanol as the solvent (2 mL). After 24 h, the enzyme was filtered off, the solvent was evaporated under reduced pressure, and the lipophenols were purified by column chromatography (SiO_2_, *n*-hexane–acetone as the eluent). Spectroscopic data of the purified products were in agreement with those available in the literature [35,36], and purity (>98%) was assessed by HPLC.

### 2.3. Cell Culture

Pancreatic β-cell line INS-1 832/13 was kindly provided by C. Newgard (Duke University, Durham, NC, USA). The insulin-secreting INS-1 832/13 is a cell line stably transfected with a plasmid coding for human proinsulin. The cells were maintained in RPMI-1640 medium supplemented with 10% fetal bovine serum (FBS), 10 mM HEPES, 2 mM l-glutamine, 1 mM sodium pyruvate, 50 mM β-mercaptoethanol, 100 IU/mL penicillin and 100 IU/mL streptomycin. Cells were incubated under 95% O_2_ and 5% CO_2_ at 37 °C (Heracell CO_2_ Incubators—Thermo Fisher Scientific) [37]. Media were refreshed every 2–3 days and cells were trypsinized and passaged weekly. Cells were sub-cultured when they achieved ≥70% confluence. Cell were used up to 12 passages after initial thawing. 

### 2.4. Glucose Stimulated Insulin Secretion (GSIS) and Insulin Detection

A total of 5 × 10^5^ INS-1 832/13 β-cells were plated in 24-well plates with RPMI 1640, 11 mM glucose, and 10% FBS. The media were switched to 5 mM glucose, and after 16 h incubation, β-cells were washed and a secretion medium (Hank’s balanced salt solution with 20 mM HEPES and 1% bovine serum albumin, pH 7.2; HBSS) containing 3 mM glucose was added to the cultures. After 2 h, the secretion medium was replaced with a fresh secretion medium containing 23 mM glucose to initiate the GSIS (at 0; 5; 15; 30; 60 and 120 min), and concomitantly cells were treated with HtyOle or TyOle at different concentrations (ranging from 10 to 40 μM) or left untreated. A separate series of experiments was performed exposing or not exposing INS-1 832/13 β-cells to HtyOle, TyOle, Ty, or Hty at a fixed concentration of 10 μM. Then, 8 µM DC260126 [38] was added to the culture media 30 min before starting the GSIS, and it lasted until the GSIS was performed. The samples were then collected and spun for 5 min at 2500 r.p.m. and at 4 °C to pellet down cellular debris. The supernatant was then stored at −80 °C until assayed. Levels of insulin were detected by a commercial ELISA kit (Calbiotech, Inc., El Cajon, CA, USA) according to manufacturer’s instructions. Each sample was run in triplicate. The insulin secretion data are presented only at 5 min. A Multi-Mode Microplate Reader (Synergy H1 Hybrid-BioTek, Winooski, VT, USA) was used to measure ELISA absorption. 

### 2.5. Insulin Immune-Cytochemistry

A total of 8 × 10^4^ INS-1 832/13 β-cells were seeded in 24-well plates on glass slides and cultured in RPMI 1640, 11 mM glucose, and 10% FBS. The media were switched to 5 mM glucose and after 16 h incubation, INS-1 832/13 β-cells were washed and a secretion medium (HBSS) containing 3 mM glucose was added to the cultures. After 2 h, cells underwent GSIS and were exposed or not exposed to 17 μM HtyOle or 21 μM TyOle, as described. Samples were then washed 2 times with PBS and incubated with a primary anti-insulin antibody (1:200, Dako, Agilent Technologies, Santa Clara, CA, USA) overnight at 4 °C, followed by three 5 min washes in PBS. Dylight 488 secondary Ab (Jackson Immunoresearch, West Grove, PA, USA) incubation was conducted for 1 h at room temperature. Samples were then washed three times and coverslips were mounted using an antifade mounting medium with DAPI (Life Technologies, CA, USA). Images were viewed at 40× magnification and captured through Leica AF6000 microscope (Leica, Wetzlar, German).

### 2.6. Computational Details

Molecular docking simulations were performed on the structure of FFAR1 (resolution 2.33 Å, protein data bank entry 4phu) [39] co-crystallized with the partial agonist TAK-875, following a computational protocol previously described [40]. Based on the structural properties of the allosteric and the inter-helical binding sites on FFAR1, and even more on the structure of ligands that were co-crystallized within them (as an example, TAK875 in the allosteric site, and an oleoyl-glycerol molecule in the inter-helical crevice), we hypothesized that tyrosyl oleates could bind to the inter-helical binding site instead of the allosteric binding site. Consequently, 4phu and 5tzr that showed the same ligand (namely, an oleoyl-glycerol molecule) at the inter-helical binding site were considered equivalent for docking simulation and, finally, 4phu was arbitrarily chosen.

The structure of ligands was sketched with Maestro software and prepared with the LigPrep routine (implemented within the Schrödinger Suite 2011) (Schrödinger, LLC. www.schrodinger.com, accessed on 21 January 2021) by application of the OPLS 2005 force field. Epik 2.2 was also used to generate tautomers at pH 6–8.

The three-dimensional coordinates of the complex between FFAR1 and TAK-875 were refined by means of the Protein Preparation Wizard by adding hydrogens and assigning bond orders. Next, potential binding sites on FFAR1 were identified with SiteMap 2.5 [41]. Finally, Glide 5.7 [42] was used for docking simulations: each potential binding site on FFAR1 was embedded into a grid that codified the physico-chemical properties of such a portion of the FFAR1 receptor. Moreover, hydroxyl groups contained within the gridbox were allowed to rotate to find optimal interactions with receptor amino acid side chains during docking calculations.

### 2.7. Statistical Analysis

Data were expressed as mean ± standard deviation from three independent experiments in triplicate. Statistical differences were determined by a one-way analysis of variance (ANOVA) followed by Tukey’s multiple comparison test as post hoc. Differences were considered statistically significant for *p* < 0.05 (*), *p* < 0.01 (**), and *p* < 0.001 (***).

## 3. Results

### 3.1. Insulin Secretion from INS-1 832/13 β-Cells under HtyOle and TyOle Treatment

**Figure 1:**
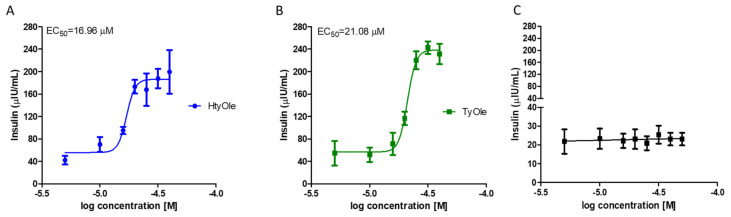
Concentration-response curves of insulin under GSIS in pancreatic INS-1 832/13 β-cells exposed to HtyOle (**A**), TyOle (**B**) and oleic acid (OA, **C**).

To perform a concentration–response curve, different concentrations of HtyOle or TyOle ranging from 0 to 40 µM were used. INS-1 832/13 cells were exposed to these molecules for 5 min under GSIS condition and insulin levels were measured in culture conditioned media by two-site immunoassay. As shown in Figure 1 panel A and B, both molecules led to a dramatic increase in insulin secretion by 5-fold for HtyOle and 6-fold for TyOle.

**Figure 2:**
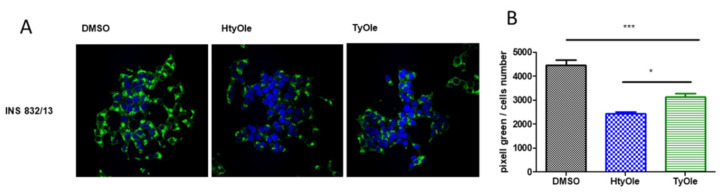
Insulin immunostaining of INS-1 832/13 β-cells, treated with DMSO (as vehicle), 17 μM HtyOle or 21 μM TyOle (**A**). Figures are representative of three independent experiments. (**B**) Pixel immunostaining analysis. *** *p* < 0.001, * *p* < 0.05. One-way analysis of variance followed by Tukey’s multiple comparison test as post hoc.

The effects of HtyOle and TyOle on the hormone levels were concentration-dependent, and the maximal effectiveness was found at concentration of 30 μM for both compounds (Figure 1A,B). The higher concentration of 40 μM was not able to further enhance insulin secretion (Figure 1A,B) for either compound. EC_50_ values were 16.96 and 21.08 μM for HtyOle and TyOle, respectively. We also tested OA at concentrations up to 50 µM without recording any change in insulin secretion (Figure 1C). To further confirm the secretagogue activity of these compounds, immune-cytochemistry by the fluorescent technique was performed, and revealed that INS-1 832/13 cells were stained positively with an anti-insulin antibody. A decrease in insulin immune reactivity was observed in the cell culture exposed to 17 μM HtyOle or to 21 μM TyOle for 5 min during the GSIS experimental condition (Figure 2A), indicating that these compounds amplified the glucose-mediated release of insulin. Quantitative analysis showed a twofold and threefold decrease in insulin immune reactivity in HtyOle- and TyOle-treated cells, respectively, compared to control cultures. The difference was highly significant (Figure 2B) and allowed us to classify them as novel insulin secretagogue molecules.

### 3.2. Pharmacodynamic Profile of HtyOle and TyOle

**Figure 3:**
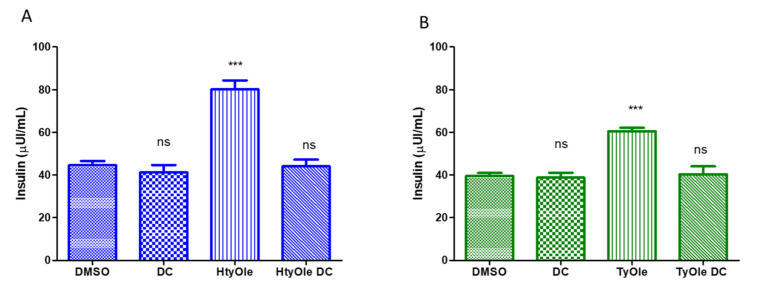
Functional assays on INS-1 832/13 β-cells exposed 8 μM DC260126 (DC) 30 min before treatment with 10 μM HtyOle (**A**) or to 10 μM TyOle (**B**) upon GSIS condition. Figures are representative of three independent experiments. *** *p* < 0.001. One-way analysis of variance followed by Tukey’s multiple comparison test as post hoc.

To better characterize the pharmacodynamic profile of these molecules, INS-1832/13β-cells were exposed to a fixed concentration (i.e., 8 μM) of DC260126, a known antagonist of FFAR1, 30 min before treatment with HtyOle or TyOle at a fixed concentration of 10 μM upon the GSIS condition. As shown in Figure 3A,B, the exposure of INS-1832/13 β-cells to DC260126 affected the facilitatory effect on GSIS, leading to a dramatic decrease in the hormone secretion compared to the cell culture treated with HtyOle or TyOle alone. In the same condition, we also tested the vehicle alone (DMSO), revealing that 35.42 ± 5.67 µIU/mL of insulin had been released.

**Figure 4:**
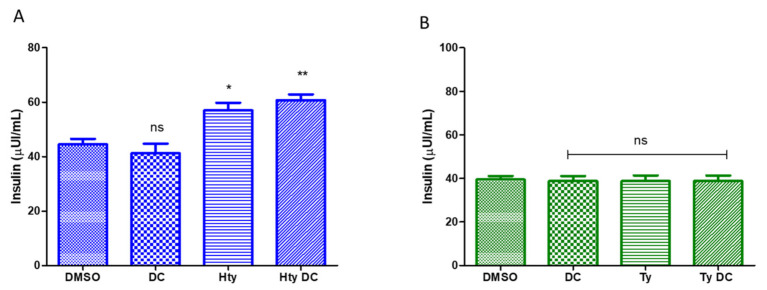
Functional assays on INS-1 832/13 β-cells exposed 8 μM DC260126, 30 min before treatment with 10 μM Hty (**A**) or to 10 μM Ty (**B**) upon GSIS condition. Figures are representative of three independent experiments. ** *p* < 0.01, * *p* < 0.05. One-way analysis of variance followed by Tukey’s multiple comparison test as post hoc.

To prove that the presence of an oleic acid chain is the major determinant in conferring a pharmacodynamic advantage, the insulinotropic effect of HtyOle and TyOle precursor was also assayed. For this purpose, INS-1-832/13 β-cells were exposed, under GSIS condition, to Hty or Ty at a fixed concentration of 10 µM, according to previous data [31]. The results (Figure 4 panels A and B) reveal that while Hty positively affected the hormone glucose stimulated secretion, Ty did not induce any change in insulin release in the culture medium.

Of note, the exposure of INS-1 832/13 β-cells to DC260126 did not influence the enabler effect of Hty on GSIS (Figure 4 panel A), indicating that the insulin secretagogue activity of this compound is not mediated by FFAR1.

### 3.3. FFAR1 Docking Simulation for HtyOle and TyOle

**Figure 5:**
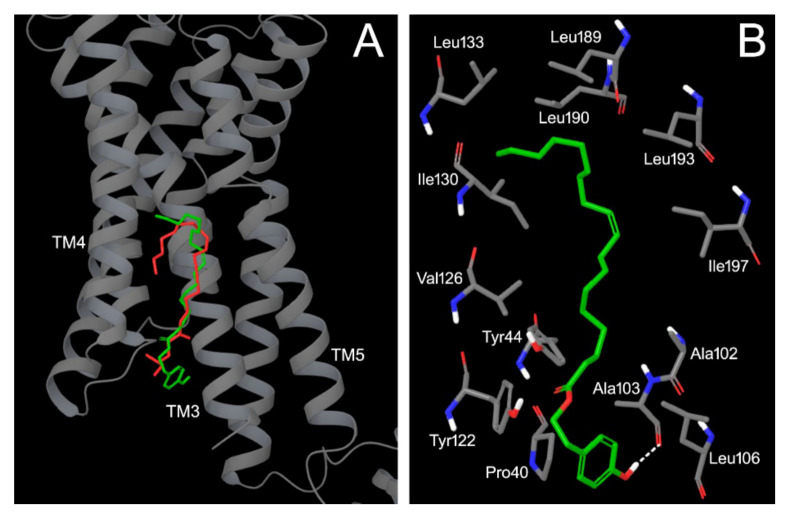
(**A**) Graphical representation of the helical motif of FFAR1 (grey) with the co-crystallized ligand (orange) and the tyrosyl oleate (green) in its best docked conformation; (**B**) close-up of the interactions between FFAR1 (atom type notation with grey carbon atoms) and the tyrosyl oleate (atom type notation with green carbon atoms), as found in the best docked pose.

To study the possible interaction of HtyOle and TyOle on FFAR1, docking simulations were performed. As an example, the long hydrophobic chain of (*2R*)-2,3-dihydroxypropyl (9*Z*)-octadec-9-enoate (the oleoyl glycerol moiety) co-crystallized with FFAR1 (entry 4phu of the protein data bank) is accommodated within a groove rich in apolar amino acids, while the terminal hydrophilic ester portion is oriented toward a cage populated by hydrophilic and positively charged residues, such as His and Arg (Figure 5A). In a similar way, the best ranked pose of TyOle showed an identical orientation within the FFAR1 binding site. In fact, the oleoyl portion is partially superimposable to the same chain of the co-crystallized ligand, and the ester groups of the two modulators are accommodated within the same region. The terminal tyrosyl moiety is folded within the trans membrane (TM) TM3-TM4 inter-helical space between Tyr122 (TM4) and Ala103 (TM3). An important anchor point is represented by a hydrogen bond between the phenolic group of the TyOle and the backbone carbonyl of Ala103 (Figure 5B). The hydrophobic tail of TyOle was accommodated within the groove mainly constituted by apolar amino acids, such as Ala, Leu, Ile, and Val (Figure 5B). In the case of the HtyOle, the ligand underwent a slight translation toward ICL2 that allowed its 3-OH substituent to make a hydrogen bond with the backbone carbonyl moiety of Ala103 (the same anchor point found for the TyOle).

These results suggest that both the HtyOle and TyOle could be located within the long and hydrophobic extrahelical binding site delimited by TM3-5 and ICL2, already found to accommodate FFAR1 synthetic and natural agonists.

## 4. Discussion

The insulin secretagogue potential of HtyOle and TyOle was studied using the INS-1 832/13 cells, as in the vitro β-cell model. This cell line is a genetically modified INS-1 cell subclone selected for its robust glucose responsiveness over the physiological range of glucose concentrations (3–23 mM) retaining a differentiated cell phenotype for over more than 6 months in culture [37]. These biological features have made it a widely used tool for studying pancreatic β-cell function and for compound-screening purposes. Although naturally occurring, in our study, synthetic Hty, HtyOle, and TyOle, and commercial standard Ty were utilized to avoid the issues related to the extraction procedure from olive oil, such as contamination or low extraction yield, thus ensuring reproducible data.

The comparison of the EC_50_ values obtained from the concentration–response curve for HtyOle and TyOle suggested a similar potency of insulin secretion. This was further confirmed by a decrease in insulin immune reactivity in the cell culture exposed to HtyOle or TyOle at EC_50_ concentration. The amount of HtyOle in EVOOs has been previously quantified as 4.9 mg kg^−1^ of oil [32], while TyOle was found in the range 0.17–1.18 mg kg^−1^ of oil, depending on the cultivar of the plant from which the olive oil originates [33]. The anti-diabetic effects of olive oil have been associated with the high content of OA [7,8,9,10], as well as with its phenolic fraction [25,26,27,43]. On the basis of the effects observed in this study, HtyOle and TyOle may be partly responsible for the anti-diabetic effects associated with olive oil consumption.

The characterization of their pharmacodynamic profile, exposing INS-1832/13 β-cells to a fixed concentration (i.e., 8 μM) of DC260126, a known antagonist of FFAR1, strongly suggested that the effect of both HtyOle and TyOle on insulin release was due to an agonist activity on GPR40 and demonstrated for the first time a novel biological property of these compounds previously categorized as antioxidant and anti-inflammatory molecules [31,35,36,38,44]. To further strengthen the hypothesis that the presence of an oleic acid chain as the major determinant in conferring a pharmacodynamic advantage, the insulinotropic effect of HtyOle and TyOle precursors was also assayed. Our results demonstrate that while Hty positively affected the hormone glucose stimulated secretion, Ty did not induce any change in insulin release in the culture medium. This finding is in agreement with recent observations, indicating a role for Ty in beta cell survival and in insulin biosynthesis, rather than on the GSIS [26]. Of note, the exposure of INS-1 832/13 β-cells to DC260126 did not influence the enabler effect of Hty on GSIS, indicating that the insulin secretagogue activity of this compound is not mediated by FFAR1 and in this respect, inhibition of K/ATP channels and the subsequent opening of the voltage-dependent calcium channel by Hty has been proposed [45].

Previous literature reports have shown that oleoyl esters [46,47] and synthetic small molecules [48,49] bind FFAR1 within a long, open, extra-helical crevice delimited by trans-membrane helixes TM3-5 and the intracellular loop 2. Our results suggest that both HtyOle and TyOle could be located within the long and hydrophobic extrahelical binding site delimited by TM3-5 and ICL2, already found to accommodate FFAR1 synthetic and natural agonists. Several small molecules have been synthesized and assayed but failed clinical trials because of serious concerns regarding their safety profile. In this context, hybrid molecules bearing a tail of oleic acid, which is an endogenous FFAR1 ligand, represent an alternative strategy for the development of FFAR1 agonists. Our previous studies have shown how this approach conferred a pharmacodynamic advantage and led to the synthesis of quercetin-3-oleate derivative working as a full or partial agonist of FFAR1 [46,47]. Functional and molecular docking approaches have highlighted HtyOle and TyOle as natural modulators of the FFAR1 receptor, confirming the idea that drugs able to affect glucose levels can be obtained by combining the structures of two naturally occurring substances. In addition, the presence of a phenolic moiety in these molecules, along with the low concentrations required for insulin secretagogue effects, could make lipophenols potential new leads. However, lipophenols should undergo rigorous lipotoxic evaluation to avoid the limitations that hampered the use of FAs as antidiabetic agents [50,51]. Moreover, we have not observed any change in insulin secretion in our experimental set-up under OA treatment. This is not in contrast with the data reported in the literature. In fact, Hu et al. observed that OA treatment (100 µM) was able to increase FFAR1 mRNA at 6 and 48 h, without measuring insulin secretion [38]. In addition, Gravena et al. showed that insulin secretion was induced by an acute dose (500 µM) of a series of fatty acids not including OA [52]. Fujiwara et al. found that OA at 50 µM significantly increased insulin release from isolated rat islets in the presence of 8.3 mM glucose, without mimicking the GSIS condition [22]. Furthermore, Hauke et al. measured the content of endogenous fatty acids released from membrane phospholipids in which OA was present at ng amounts [53]. On the other hand, both Hty and Ty have been shown to preserve β-cell function and survival through the activation of anti-inflammatory and antioxidant pathways [26]. Our future research directions will be devoted to the preparation of structurally related and modified lipophenols, in which the phenolic OH groups could be replaced by one or two methoxy groups.

## 5. Conclusions

Glycaemic control is of key importance in the successful management of T2DM, but despite the availability of glucose-lowering drugs with different pharmacodynamic profile, reversing the disease process and restoration of normal glucose homoeostasis rarely occurs. We found that HtyOle and TyOle displayed insulin secretagogue activity in pancreatic β-cell line INS-1 832/13 under GSIS condition. Remarkably, this effect was observed at relatively low concentrations. Functional pharmacological approaches and molecular docking simulation support the involvement of free fatty acid receptor 1 (FFAR1) in the observed bioactivity for both lipophenols. Taken together, our results suggest that the anti-diabetic properties associated with olive oil consumption can be partly due to the occurrence of HtyOle and TyOle.

## Data Availability

Data are contained within the article.
